# Sub-Optimal Allocation of Time in Sequential Movements

**DOI:** 10.1371/journal.pone.0008228

**Published:** 2009-12-09

**Authors:** Shih-Wei Wu, Maria F. Dal Martello, Laurence T. Maloney

**Affiliations:** 1 Department of Psychology, New York University, New York, New York, United States of America; 2 Division of the Humanities and Social Sciences, California Institute of Technology, Pasadena, California, United States of America; 3 Department of General Psychology, University of Padova, Padova, Italy; 4 Center for Neural Science, New York University, New York, New York, United States of America; Massachusetts Institute of Technology, United States of America

## Abstract

The allocation of limited resources such as time or energy is a core problem that organisms face when planning complex actions. Most previous research concerning planning of movement has focused on the planning of single, isolated movements. Here we investigated the allocation of time in a pointing task where human subjects attempted to touch two targets in a specified order to earn monetary rewards. Subjects were required to complete both movements within a limited time but could freely allocate the available time between the movements. The time constraint presents an allocation problem to the subjects: the more time spent on one movement, the less time is available for the other. In different conditions we assigned different rewards to the two tokens. How the subject allocated time between movements affected their expected gain on each trial. We also varied the angle between the first and second movements and the length of the second movement. Based on our results, we developed and tested a model of speed-accuracy tradeoff for sequential movements. Using this model we could predict the time allocation that would maximize the expected gain of each subject in each experimental condition. We compared human performance with predicted optimal performance. We found that all subjects allocated time sub-optimally, spending more time than they should on the first movement even when the reward of the second target was five times larger than the first. We conclude that the movement planning system fails to maximize expected reward in planning sequences of as few as two movements and discuss possible interpretations drawn from economic theory.

## Introduction

A central concern shared by microeconomics, behavioral ecology, psychology, and neurobiology is how well organisms allocate limited resources. For economists, knowing how buyers allocate their financial budgets is essential for understanding consumer behavior [1]. In animal foraging, behavioral ecologists seek to formalize how animals allocate time and energy constraints to maximize survival [2].

There are several previous studies showing that human subjects can adjust the duration of *single* movements so as to maximize reward or nearly so [3]–[5]. However, almost all previous studies of movement planning have focused on planning just one reach or grasp [6]–[14]. Many everyday tasks consist of discrete movements carried out in a sequence. Very few studies, however, have investigated sequential movement planning [15], [16].

In this study, we examined human ability to allocate time in more complex tasks that model the kinds of tradeoffs we make every day. In these tasks, the subject can invest more or less time in any of several activities in succession but the total amount of time available is fixed. Solving this sort of problem falls within the domain of optimal search theory [17] and statistical decision theory [18], [19]. We have translated this sort of problem into an experimental design to investigate human ability to allocate time among successive movements.

**Figure 1:**
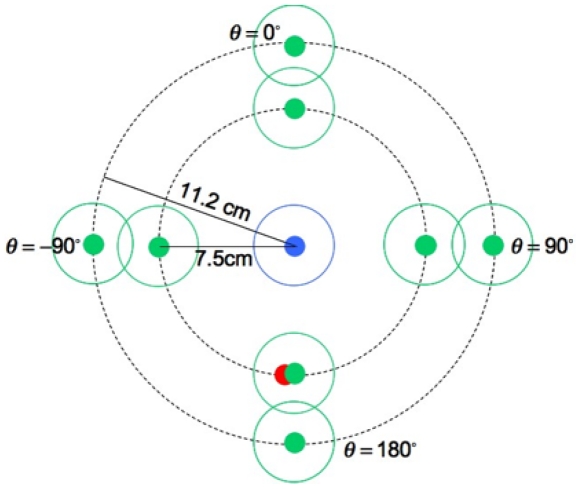
Sequential movement task. In a visually guided sequential pointing task, subjects started every trial by placing their index finger on the starting position (red dot). The subject's task was to hit the blue target (referred as target A in the main text) and the green target (target B) in sequence within 400 ms. The green target was located in one of the eight possible locations as shown. The eight possible locations of the green target were determined by the four possible angle changes (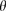) between the first and second movement and the two possible distances between the first and second targets. We emphasize that only one green target was present on each trial.

In our experiment, subjects had a very limited time window (400 ms) to complete two successive reaching movements to two targets in a specified order. A schematic of the task can be seen in Figure 1. At the start of each trial subjects first placed their finger on a red dot and, as soon as they were ready, moved rapidly to touch a blue target and a green target in that order. In this experiment, we manipulated the change of direction between movements, movement distance, and the rewards assigned to each of the targets. The blue target was always at the center. The green target could be at any of eight locations as shown but only one green target was present on each trial. Subjects could take as much time as desired to plan their movements before initiating movement, but, once they began moving, they had only 400 ms to complete both movements.

We wanted to force subjects to at least attempt to hit both targets on each trial. Accordingly, we marked a larger circular region around each target. Subjects knew that, if they did not touch within both large circular regions on a trial, they would receive no reward for any targets they hit on that trial. See *Materials and Methods* for details.

If subjects completed their movements within the time limit and hit within both large circles around the targets, they received a monetary reward for each target they actually touched. We varied the monetary rewards associated with the green targets, the location of the green target, and the distance between the blue and the green targets (See *Materials and Methods* for details). Subjects always knew the potential rewards associated with each of the two targets and the locations of both targets before the start of each trial. To avoid any effect of concurrent feedback on the movements, visual feedback specifying which targets were hit was provided only after the termination of the second movement (see [15] for a discussion on the effect of concurrent feedback on sequential movements).

### A Model of Optimal Sequential Movement Planning

We developed a model of optimal time allocation based on statistical decision theory [18], [19] and previous work on movement planning [8], [9]. The full model is described in *Materials and Methods*. Here we lay out critical concepts and intuitions necessary for the readers to understand the model. We use the terms “gain” and “reward” interchangeably, and we used the terms “optimal” and “ideal” to describe behavior maximizing expected gain.

Our goal was to predict the allocation of time among movements that maximizes the subject's expected gain. We use this criterion (maximization of expected gain) as a benchmark and we compare human performance to this benchmark. This sort of comparison has a celebrated history in the study of perception and action (see e.g. Geisler [20], Ernst & Banks [21]). This class of models is central to optimal foraging theory (behavioral ecology) [2] and serves as benchmarks in economic decision making (maximum expected gain, maximum expected utility) [1]. In considering any human performance it is natural to first ask how close performance comes to maximizing expected gain or expected utility.

**Figure 2:**
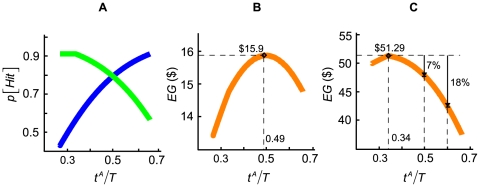
Maximizing expected gain. **A.** The probability of hitting the first target A (blue) and the second target B (green) for subject YCC were plotted as functions of the proportion of time allocated to the first movement (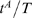). As more time was spent on the first movement, the probability of hitting the first target increased, while the probability of hitting the second target decreased. **B.** The sum of the expected gain of the two targets was plotted as functions of 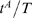. Here, the reward for hitting the first and the second target were bot $10. The maximum on the orange curve ($15.90) corresponded to 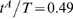. **C.** The same format as Figure 2B, but now hitting the first target earns a reward of $10 while hitting the second earns a reward of $50. Compared with the condition where the target rewards were equal, the maximum on the orange curve ($51.29) has shifted to the left with 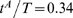, indicating that subject YCC should allocate more time on the more rewarding target in order to maximize expected gain. The vertical arrows represent the loss to the subject that results from allocating time non-optimally expressed as a percentage of maximum expected gain.

The intuition behind the model is the following: the more time the subjects spend on moving to a given target, the more likely they are to hit it. This is a consequence of speed-accuracy tradeoff (SAT). Due to the fixed total time constraint, the tradeoff in time between the two movements introduces a tradeoff in accuracy between the two movements. In Figure 2A, we plot examples of SAT curves describing the probability of hitting the first target 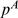 (blue) and the probability of hitting the second target 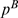 (green) as functions of the proportion of time allocated to the first target 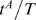. 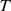
denotes the mean of total movement time: movement time to the first target A plus movement time to the second target B. Since each subject typically had a different value of 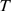, we express time in terms of relative time 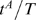.

The probability 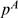 is plotted as a blue curve that increases when the time spent on the first target increases. The probability of hitting target B, 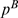, is plotted as a green curve that *decreases* when the time spent on the first target increases since time allocated to the first movement comes at the expense of time allocated to the second. This plot captures the tradeoff in accuracy between the two movements as a function of how total time is divided. Although we present Figure 2 as a hypothetical example, it is based on actual data from one subject.

Now, suppose the subject receives a reward of $10 for each target hit. We compute expected gain for target A (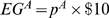) and expected gain for target B (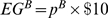) as functions of 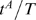. At any 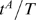, the total expected gain from both targets in a trial is just the sum 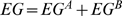. It is plotted in orange in Figure 2B as a function of 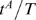.

When the target rewards are equal (Figure 2B), 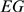 is at its maximum ($15.90) when 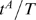 is about 0.49. What about when the target rewards are not equal? In Figure 2C we compute expected gain as in Figure 2B, but now the first target is worth $10 and the second is worth $50. The second target is five times as rewarding as the first one and the optimal 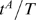 shifts to about 0.34. By spending less time on the first movement, the subject can spend more time on the second, more rewarding movement and the maximum expected gain from both targets together is now $51.29.

In Figure 2C we also illustrate the loss in expected gain resulting from allocating time non-optimally by vertical arrows together with the reduction in expected gain expressed as a percentage of the maximum.

### Estimating Speed-Accuracy Tradeoffs for Multiple Movements

Figures 2BC capture the qualitative predictions of the model. In order to develop quantitative predictions concerning optimal time allocation we need to be able to predict the SAT curves in Figure 2A for each condition and for each subject. Accordingly, we developed and tested a model of SAT for two successive movements that is a natural extension of existing SAT models for single movements [22]–[27]. This model allowed us to predict the allocations of time that maximized expected gain for each condition and to compare human performance to ideal.

### Results Summary

The results of the experiment were as follows.

First, we found clear evidence that the outcomes of the two movements in succession were statistically independent, each depending only on the time invested in the corresponding movement. This allowed us to model the two movements as independent, linked only by the constraint that more time allocated to the first movement left less time for the second.

Second, in estimating the SAT, we found that movement error along the direction of movement increased more rapidly as a function of speed than that orthogonal to the movement direction.

Third, in contrast to our expectations, we found clear, qualitative failures in subjects' allocation of the fixed time budget across the two successive movements. We emphasize that the subject was allowed as much time as desired to plan the two movements on each trial; timing did not start until the subject began to move. Nevertheless, subjects did not allocate appreciably more time to the movement towards the more valuable of the two targets even when the ratio of target values was as extreme as five to one.

We conclude that, while single movements may be planned optimally (or nearly so), as past research indicates, the movement planning system fails to maximize expected gain in planning sequences of as few as two movements, a limitation on movement planning that could be construed as a form of bounded rationality [28], [29]. We discuss possible connections with temporal discounting in economics [30], [31] and the possible effects of training.

## Results

We first tested hypotheses needed to develop a model of the subject's movement error and speed-accuracy tradeoff. The outcomes of these tests allowed us to formulate an accurate model of SAT for each of two successive movements. Given this model, we could then compare human performance to ideal performance maximizing expected gain.

### Movement Independence

In our task, subjects made two successive movements, allocating time to each. We varied the change in movement direction between the successive movements (4 conditions), movement distance (2 conditions), and reward profiles (2 conditions). Hence, there were a total of 16 conditions. See *Materials and Methods* for details. Given a fixed total time constraint, the first question we were trying to address was, would the trial-to-trial spatial variability in the first movement affect the outcome in the second? In other words, would errors propagate from the first movement to the second? The second question was whether the probabilities of hitting the two targets were statistically independent, affected only by the time allocated to each movement. We therefore tested independence in two ways.

First, we examined the correlation between the first and second movement endpoints. For each subject, we computed the correlation separately for the x and y directions and for each condition and found no correlation in most cases (*p*-value Bonferroni-corrected at 0.0015 for 32 total conditions for each subject). Across subjects, only 2 out of 32 conditions on average had correlations significantly different from 0. This outcome indicates that the first and second movements conditional on the time allocated to each were effectively independent for almost all conditions.

**Figure 3:**
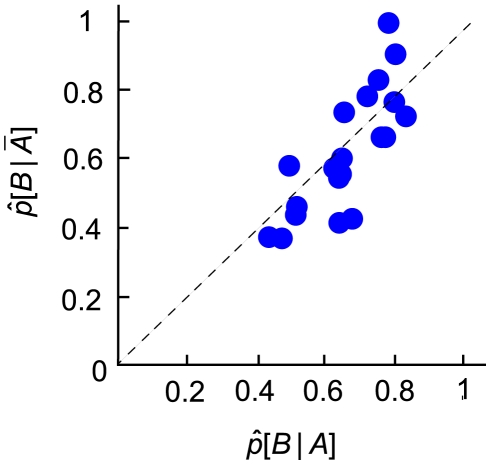
Statistical independence of movements. The probability of hitting the second target given that the first target was missed 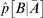 was plotted against the probability of hitting the second target when the first target was hit 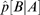. Each point represented a combination of reward and distance conditions from a subject. As most points are distributed symmetrically about the diagonal line, the outcomes of the two movements can be treated as statistically independent. See text.

We then performed a second analysis to test statistical independence of the outcome of the two movements. For each distance and reward condition (See *Materials and Methods* for details on the design), we computed the proportion of times the subject hit target B (the second target attempted) after hitting target A (the first target), denoted 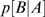, and the proportion of times the subject hit target B after missing target A, denoted 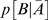. The graph in Figure 3 plotted estimates of 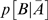 against estimates of 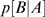 across subjects. Each point represented a combination of distance and reward condition for a subject. If the two movements were independent, hitting or missing the first target should not affect the chance of hitting the second target. Hence, we would expect each point to fall on the diagonal line. Qualitatively, this was what we observed across subjects, as most points lie close to the diagonal line.

We also attempted to examine independence quantitatively. It is obvious that the distance between each data point and the diagonal line indicates the extent of deviation from independence. The greater the distance is, the more the two movements deviate from independence. We used a bootstrap method to obtain confidence intervals [32]. For each point on the graph, we resampled the corresponding data 10,000 times to compute the confidence interval for the distance. None of the points significantly deviated from the diagonal line (p>0.0125, Bonferroni corrected for the number of combinations of conditions for each subject).

We concluded that, the two movements on each trial, at least in terms of hitting or missing the targets, could be modeled as two statistically independent movements, linked only by the constraint on the time allocated to each movement. This independence allowed us to develop a simple model of optimal allocation described in *Materials and Methods*.

### Movement End Points

We next verified that the distribution of movement end points was close to bivariate Gaussian as found in previous work [33]. Second, we tested whether subjects aimed at the center of the targets. We rejected the hypothesis that subjects aimed at the center of the target (aim point assumption) for all 5 subjects in at least one condition. However, the estimated deviations were small compared to the size of the target and the size of the finger pad. The radius of the target was 5.88 mm. The mean deviation in the horizontal direction across subjects and conditions was 0.0518 mm, and 0.7182 mm in the vertical direction. These small failures may simply reflect a difference between what the touch screen records as the end point of a movement and what the subject considers to be the end point. In “Estimating speed-accuracy tradeoff (SAT)” below, we compared subjects' probability of hitting the targets to that predicted by the model with the aim-point assumption. We found that these small deviations had negligible effect on subjects' estimated probabilities of hitting targets. See also the discussion related to *Estimating speed-accuracy tradeoff*.

### Estimating Speed-Accuracy Tradeoff (SAT)

As described in the Introduction, we modeled SAT for sequential movements based on past work [22]–[27] for single movements. We assumed that the standard deviation (accuracy) of movement error increased as a linear function of average speed. We denoted accuracy as 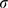. The key to estimating SAT here is to model the SAT for movements separately in 2 orthogonal directions. We separately computed the standard deviation of movement end points parallel to the direction of movements, denoted 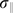, and the standard deviation of movement end points orthogonal to the direction of movements, denoted 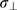. See *Materials and Methods* for details of the coordinate system we defined for movement end points.

**Figure 4:**
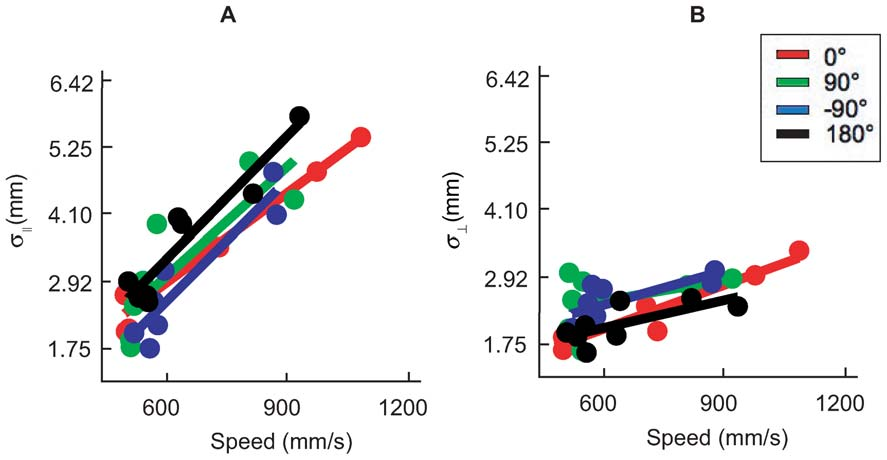
Speed-accuracy tradeoff (SAT). **A.** Spatial variability parallel to the direction of movement 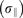 was plotted as a function of the average speed of the movement (mm/sec) from subject AI. Different colors coded for different direction conditions. Each data point represents a single condition. The lines represented the best fitted linear SAT functions (Eq. 4). **B.** Spatial variability perpendicular to the direction of movement 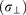 was plotted against average speed from the same subject.

For each direction condition, we first estimated 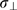 and 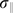 separately as a function of average speed (Eq. 4 in the model section under *Materials and Methods*). Direction here refers to the change in movement direction between movement to the first target and movement from the first target to the second target. There were four possible directions (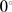,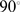,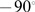,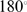) as shown in Figure 1. In Figure 4, we plotted the estimated SAT function separately for each direction from a single subject (AI). Different directions were coded with different colors. In Figures 4A, 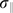 was plotted against averaged speed. In Figure 4B, 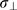 was plotted against averaged speed. In general, we observed that (1) 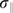 increased more sharply as a function of speed than 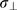 and (2) direction had negligible effect on the SAT profile. Across subjects and conditions, the mean 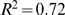 for the fit of 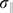, while the mean 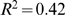 for the fit of 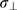. We conjecture that the lower goodness of fit revealed by 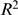 for 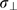 was partly due to the fact that the regression slope of 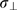 as a function of time was much shallower than that of 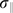, and in 12 of the 20 conditions across subjects (4 direction conditions for each subject, 5 subjects) it did not differ from 0 (*p*>0.05). The lower goodness of fit revealed by 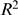 is likely due to the reduced dynamic range of 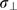 as a function of speed with random variation of 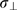 across conditions resulting in smaller 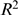.

The covariance matrix for errors parallel to the direction of movement and errors orthogonal to the direction of movement is denoted 
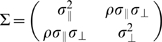
. We tested whether the correlation 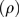 between parallel and orthogonal errors was 0. We found that across subjects, the correlation between x and y endpoints was generally not different from 0 (p>0.0016, Bonferroni corrected for the number of conditions for each subject). Three out of five subjects had no significant correlations across all conditions. For those who showed significant correlation in more than one conditions,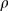 was significantly different from 0 in only one out of 32 conditions for subject AI, and in 2 out of 32 conditions for subject SAS.

If 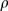 were zero in most cases, then it should not vary as a function of speed as well. An additional analysis that regressed 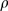 against speed was performed. The regression analysis revealed that both the slope and the intercept were not significantly different from 0 in all 5 subjects. We thus concluded that there was no correlation between x and y endpoints and hence the covariance matrix could be expressed simply as 
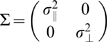
.

**Figure 5:**
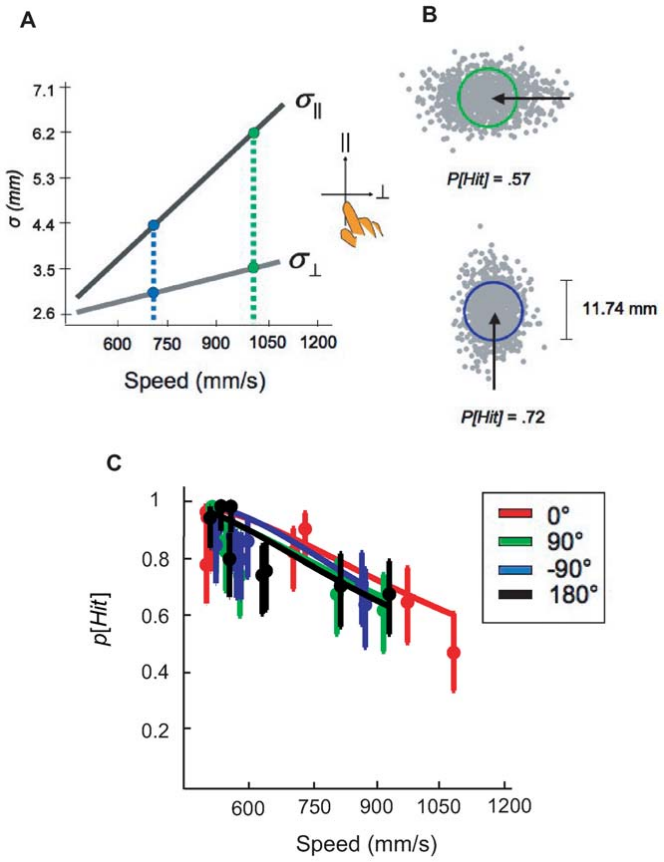
Predicting the Probability of Hitting a Target. We illustrate how we predicted the probability of hitting targets using data from one subject (AI). **A.** Suppose that the subject chooses the speed of the first movement marked in blue (710 mm/sec). Then, due to the time constraint, the speed of the second cannot be less than that marked in green (1008 mm/sec). For each movement, its speed can then be mapped onto the accuracy based on the SAT estimated from each subject and each condition. **B.** Based on the accuracy profile described in 5A, we simulated 10,000 points for each of two movements and plotted them as shown. As a consequence of speed-accuracy tradeoff, the time constraint, and the size of the targets, the probability of hitting the first was .72 and that for hitting the second was .57. The arrows represented the direction of movement. **C.** We plotted the probability of successfully touching targets estimated from the obtained speed-accuracy tradeoff with the actual proportion of the targets with 95% confidence interval as a function of average speed. Different colors coded for different direction conditions.

The results just described allowed us to accurately predict the SAT for each movement for each subject in each condition given the time allocated to the movement. In Figure 5 we illustrate simulated end points for movements of two different durations in two different directions (marked by black arrows) based on the SAT model of subject AI in Figure 4. The “spread” of end points was greatest along the direction of movement as 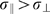 and 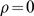.

We used the estimated SAT to predict the probability of hitting either target as a function of the time allocated to the corresponding movement. The computation was described in detail in *Materials and Methods* (see the discussion surrounding Eq. 3). In Figure 5, we illustrate how we performed this computation. Suppose that, on a particular trial, the subject moves to the first target (target A) at a mean speed of 710 mm/sec. This effectively determines the spatial variability of the first movement at 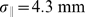 and 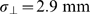 (the blue dashed line in Figure 5A) shown in Figure 5A. Due to the fixed time constraint, choosing this speed effectively determines the maximum time available for the second movement and therefore the minimum possible average speed toward target B, 1008 mm/sec. With this speed, the spatial variability of the second movement would be 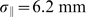 and 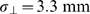 (the green dashed line in Figure 5A). We simulated this possible tradeoff 10,000 times assuming that (1) subjects aimed at the center of the targets and that (2) movement end points were distributed as bivariate Gaussian. In Figure 5B, we plotted the simulated movement end points. For each target, we estimated the probability of hitting the target by counting the number of trials where the end points fell within the target. The estimated probability of hitting the first and the second target was 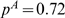 and 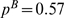 respectively.

We next verified that these predicted values matched subjects' actual performance. In Figure 5C, we plotted the estimated probability of hitting the target along with the actual fraction of hitting the target for subject AI. Different colors represented different direction conditions. If our assumptions (aiming at center and end points distributed as bivariate Gaussian) were correct and the estimated SAT were accurate, we would expect to see a close match between the estimated probability of hitting and actual performance. This was what we generally observed. We found that the estimated probability of hit matched the subjects' actual probability of hit. We also observed that direction had little effect on the probability of hitting the target and the estimated SAT. Nevertheless, we took into account these directional effects in predicting optimal performance (Eq. 5 in the model section under *Materials and Methods*).

### Model Comparison

**Figure 6:**
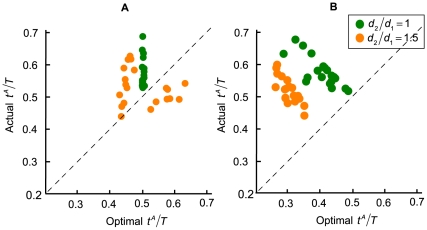
Model comparison across subjects. **A.** Equal-reward condition. In the equal-reward condition, the first and the second target had equal amount of rewards. The actual proportion of time subjects allocated to the first movement 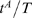 was plotted against the optimal 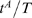. Each point represented a unique combination of distance and reward conditions. The different colors represented distance conditions, with the green representing the equal-distance condition and the orange representing the unequal-distance condition. **B.** Model comparison for the unequal-reward condition. In the unequal-reward condition, the second target was worth five times more than the first target.

We next compared actual performance to model predictions. In Figure 6, we plotted the actual proportion of time subjects allocated to the first movement, 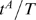, against the predicted optimal time in the two reward conditions. Figure 6A shows the model comparison when target rewards were equal. When the distance from the start position to the first target 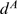 and the distance from the first target to the second target 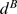 were equal (green dots), the model predicted that the subjects should allocate the time roughly equally between targets. This was not what the subjects did. Instead, we saw a tendency to spend more time on the first target even when both the movement distance and the rewards were equal between the two targets. When the second movement length increased (orange dots), the model predicted that subjects should speed up the first movement in about half of the conditions (across subjects) and slow down in the other half. This was not what we observed in our subjects.

In Figure 6B, actual performance was compared to the model prediction when the second target was five times more rewarding than the first. The model predicted that subjects should spend considerably more time on the second movement (optimal 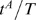 below 0.5 across all conditions and subjects). As the second movement distance became longer, the model predicted an even greater increase in the time spent on the second target, as most orange dots (coding for the unequal-distance conditions) were to the left of the green dots (coding for the equal-distance conditions).

When the second movement distance increased, subjects did increase the time spent on the second movement. However, this increase was far from optimal. We also found that across subjects and distance conditions in Figure 6B, there was a tendency to slow down the first movement when subjects should speed up and vice versa. To test for this possibility, we modeled actual performance as a linear function of model prediction and performed separate linear regressions for the equal-distance and unequal-distance conditions. In both conditions the estimated slopes of the fitted lines were negative (slope values −0.669 for the equal-distance condition and −1.177 for the unequal-distance condition) and significantly different from 0 (p<0.05), confirming that subjects changed their time allocations but in doing so they reduced rather than increased expected gain.

We wish to emphasize that by comparing actual performance with model prediction separately for each condition, we effectively controlled for confounding factors such as motivation due to difference in total payoffs between the equal-reward and the unequal-reward conditions. If subjects were more motivated in the unequal-reward condition where total payoffs were higher, they should be closer to optimal than in the equal-reward condition. We found this not to be the case, thus excluding differences in motivation due to payoff differences as a possible interpretation of the sub-optimal performance we observed.

To further demonstrate sub-optimality, we analyzed the effect of randomly speeding up the first movement on total payoff. In the unequal-reward condition where the second target's reward was 5 times greater than the first target, the model predicted that subjects should allocate much less time to the first movement. We found that, in 4 out of 5 subjects, the average payoff was slightly higher when subjects spent relatively less time on the first movement compared with trials where subjects spent more time on the first movement. Consistent with the model prediction on spending less time on the first target when the second target rewards became larger, this result indicates that even randomly speeding up the first movement could marginally improve the total payoffs. This result, along with the model comparison results explained earlier (Figure 6), clearly indicates sub-optimal performance.

### Movement Times and Dwell Time

**Figure 7:**
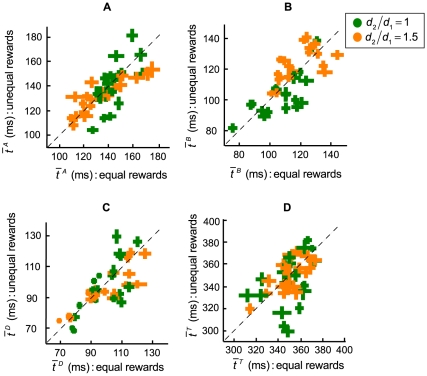
Movement times and dwell time. For each subject and condition, we plotted the mean movement times and dwell time of the unequal-reward conditions against those of the equal-reward conditions. Different colors were used to represent different distance conditions. Green represented equal-distance condition, while orange represented unequal-distance condition. **A.** Mean movement time to the first target 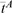. **B.** Mean movement time to the second target 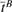. **C.** Mean dwell time 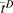. **D.** Mean total time 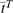. Error bars represented 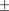
2 standard error of the mean.

In Figure 7, we reported mean movement time to the first target (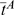), to the second target (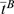), the mean dwell time (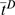), i.e. the time the finger was on the first target before initiating movement to the second target, and the mean of total time (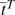) (the sum of the previous 3 time variables) from all subjects and conditions. In Figure 7A, we plotted 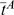 of the unequal-reward conditions against 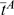 of the equal-reward conditions. Different colors (green and orange) were used to code for different distance conditions. Green represents equal-distance condition, while orange represents unequal-distance condition. Each data point represents a combination of distance, direction, and reward conditions from a single subject. By plotting the data this way, we can easily compare the effect of different reward conditions and the effect of different distance conditions on movement times and dwell time. We emphasize that the optimal solution maximizing expected gain is computed with each particular condition's mean total time, not with mean total time averaged across conditions.

If the subjects did *not* change movement times and dwell time in response to different reward conditions, we would expect all the data points to fall close to the diagonal line in all the plots. To test whether the data points deviated significantly from the diagonal line, we performed a simple regression on unequal-reward trials against equal-reward trials separately for 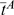, 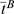, 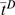, and 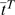. Except for 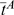, we found that the slope was indistinguishable from 1 (*p*>0.05) and that the intercept was not significantly different from 0 (*p*>0.05) for 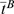, 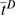, and 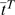. For 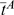, the intercept was 35 ms, suggesting that the subjects spent more time on the first target when the target rewards were unequal (the second target reward was 5 time the first target reward). This was consistent with the results in *Model Comparison* where we noted that subjects spent more time on the first target when the optimal solution was to spend less time on the first target as a result of an increase in the value of the second target.

By similar logic, if distance had no effect on movement times and dwell time, we would expect strong overlap between the distribution of green dots (indicating equal-distance) and the distribution of orange dots. We observed that subjects sped up the first movement when the second movement distance increased (7A), but distance seemed to have little effect on second movement time (7B), dwell time (7C), and total time (7D).

In addition to the plots shown in Figure 7, we reported that the mean dwell time across subjects was 97 ms. Across subjects, the maximum dwell time was 130 ms, while the minimum dwell time was 68 ms.

To summarize, the results of movement times and dwell time indicate that, first, different reward conditions did not alter movement time to the second target, dwell time, and total time. When the reward of the second target was 5 times greater than the first, subjects spent more time on the first target compared with when the rewards were equal. This result was consistent with the results reported in *Model Comparison*. Second, we found that across subjects and conditions, rewards and distance conditions had little effect on dwell time. Third, we found that across all subjects and conditions, total time (the sum of mean movement time to the first target, mean movement time to the second target, and the mean dwell time) was significantly smaller than the time limit (400ms) as it must be if the subject is to complete both movements within 400 msec on most trials.<>

## Discussion

Recent studies concerning movement planning have compared how humans plan movements to the predictions of decision-theoretic models of ideal movement that maximizes expected gain [3]–[5], [8]–[14]. Battaglia and Schrater [3] examined how humans trade off viewing time and movement time to minimize visuomotor variability; Dean, Wu and Maloney [5] looked at how humans trade off speed against accuracy in tasks where subjects were rewarded for both speed and accuracy. Together, the evidence thus far indicates a near-optimal planning system that takes into account visual and motor variability when solving the tradeoff problem (but see Wu et al. [14], Mamassian [34] and Burr, Banks & Morrone [35] for examples of suboptimal performance in perceptual and motor tasks).

Most studies to date, however, have focused primarily on investigating and modeling single movements. In this study, we investigated how humans plan sequential movements and whether they could do so optimally. In our task, two targets carrying monetary rewards were presented. Subjects had a time window of 400 milliseconds to hit the targets in pre-specified order. Subjects were required to finish both movements within the time limit but allowed to freely allocate more or less time to one movement at the expense of the other. We varied movement distance, directional change between the movements, and target rewards to further investigate how these variables could affect performance.

We extended previous work concerning SAT for single movements and used the resulting SAT model to develop an optimal model of time allocation for the sequential task. In developing the latter model, we started with the evident constraint that, the more time spent on one movement, the less time remained for the other. Given the speed-accuracy tradeoff typically observed in single movement, the accuracy of each movement is determined by the time it is taken to perform (when distance is controlled for). Taken together, the task introduced a tradeoff in accuracy between the two movements: improving the accuracy of one movement is achieved by sacrificing the time and hence the accuracy of the other movement.

In everyday life, the goal of movement often goes beyond minimizing movement variability [36], [37]. Maximizing expected gain requires that we take into account the rewards and penalties associated with different movement outcomes.

We now summarize our results. First, error along the direction of movement tended to increase more rapidly as a function of speed than error orthogonal to the direction of movement. Second, the correlation between these two directional errors was close to 0 and does not change as a function of speed. Third, we found no evidence that success in the second movement was dependent on success in the first or duration of the first, suggesting that the consequence of the two movements were independent of one another.

Based on these results, we developed a model of SAT for multiple sequential movements that allowed us to predict the optimal allocation of time for each condition and subject. We compared subjects' actual performance to ideal and found that subjects failed to divide time optimally both in conditions where the target rewards were equal and in conditions where they were not.

We found that subjects allocated more time than they should on the first movement. When the target rewards were equal, subjects could have earned more had they divided the time equally, but generally the average earning did not differ much from the maximum expected gain. However, as the second target became much more rewarding than the first, favoring the first target would lead to a marked reduction in expected gain. Comparing subjects' choice of allocation with the optimal in the unequal-reward condition (Figure 6B), we found that subjects not only spent more time on the first target, but they also tended to allocate time *contrary* to the model prediction. We observed that across subjects, the more the model predicted the subjects should favor the second target, the *less* time the subjects actually spent on the second movement and vice versa. We emphasize once again that subjects could spend as much time as they wished planning their movements before initiating the first movement: timing did not start until subjects began to move.

In the work reported here we tested whether subjects allocate total time between the two movements so as to maximize their expected gain. We tested this claim given whatever choice of total time subjects made. In any case, optimal allocation of total time between two movements is independent of the question whether the planned total time is optimal. In theory we could also test whether their choice of total time was optimal by working out the proportion of times-outs that should occur with optimal choice of total time. But estimating rates of an event with very low probability of occurrence is difficult without more data than we have. The actual time out rates (proportion of trials) are low (less than 3% in general). Moreover, this analysis would require that we accept that temporal uncertainty is Gaussian far out in the tails of the distribution where we have little data to support the distributional assumption.

Compared to previous results on human movement planning, in this study, we found a clear, patterned deviation from optimality in motor performance. We next discuss 4 possible explanations for the tendency to spend too much time on the first movement.

### The Effect of Experience

**Figure 8:**
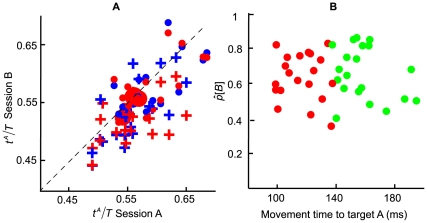
Detailed comparisons. **A.** A comparison of time allocation in the training session with the experimental session. We plotted the proportion of time subjects allocated to the first movement 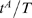 in the experimental session against that in the training session. If 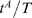 were similar between the experimental and the training session, most points would fall symmetrically about the diagonal line. Colors were used to code for the reward conditions (blue: equal-reward condition; red: unequal-reward condition). Different symbols were used to code distance conditions (dot: equal-distance condition; cross: unequal-distance condition). **B.** The estimated probability of hitting target B (second movement) (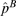) was plotted against the mean movement time (ms) to target A (the first movement) separately for the top 25% fastest movements (in red) and for the bottom 25% movements (in green). Each data point in the graph represented a combination of reward and distance condition from a subject. The duration of the first movement had little effect on the probability of success of the second movement.

It is possible that how subjects allocated time in the training session (Session A) played a role in their time allocation in the experimental session (Session B). In Session A we did not reward subjects for touching targets. Subjects were simply told to learn the time constraint and to try to hit the targets as often as they could. In Figure 8A, we compared data from Session B with data from Session A across subjects. The proportion of time allocated to the first movement 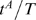 in the experimental session was plotted against that in the training session. Different symbols were used to code distance (equal or unequal), while different reward conditions (equal or unequal) coded by color. Since there were no rewards in Session A, data from the Session A was used twice to plot for the two reward conditions in Session B. Overall, the majority of the subjects spent more time on the first target in the training session as most points fell between 0.5 and 0.7. The subjects actually allocated the time very similarly in the training and the experimental session. It remains to be seen if subjects would perform better in situations where they had to spend less time on the first target had we explicitly trained them to vary their time allocation in the training session.

### The Possible Benefit of Slowing Down the First Movement

It is also possible that slowing down the first movement improves the accuracy of the second movement. Hence, increasing the first movement time marginally might be beneficial to the second movement. To analyze this conjecture, in Figure 8B, we plotted the probability of hitting target B (second target), 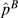, as a function of the mean movement time (ms) to target A (first target) separately for the top 25% (fastest) first movements (in red) and the bottom 25% (slowest) movements (in green). Each data point represents a unique combination of reward and distance condition from a subject. If there were a benefit of marginally slowing down the first movement on the probability of hitting the second target, we should expect to see, across conditions and subjects, that 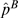 is an increasing function of mean movement time to target A. We did not find this to be the case (*r* = −0.002, p>0.05 when tested against *r* = 0). This graph also demonstrated the lack of tradeoff in time between the two movements. If subjects were optimal, we should see that increasing the speed of the first movement should improve the probability of hitting the second target. We did not observe this effect in Figure 8B, a further indication that subjects did not trade off time.

### The Possible Benefit of Off-Center Aiming

Although our model explicitly claimed that aiming at the center is the optimal aiming strategy, the model allows for off-center aiming. Here we ask whether aiming slightly off center of the first target towards the second target would be a better strategy than aiming at the center. The reasoning is the following: by aiming off-center for the first target toward the second, there is a slight reduction in movement distance to the second target and hence a possible increase in the probability of hitting the second target. We analyzed the possible benefit of this strategy and found that under the conditions of our experiment, the cost (reduction in probability of hitting target A) was very large compared to the benefit gained by reducing the length of the movement to target B. Through simulations, we found that moving 5 mm (roughly the target radius) away from the center of the first target towards the center of the second target would typically result in a 25%–30% decrease in the probability of hitting the first target but only improved the probability of hitting the second target by 5%. Given that the targets were very small (5.88 mm radius) compared with the movement distances (7.5 cm and 11.2 cm), it is obvious that any shift in aiming would have a large impact on the probability of hit but would only reduce slightly the movement distance to the subsequent target.

### The Utility of Movements and Temporal Discounting

One possible explanation for what we have observed is that subjects preferentially valued the targets by the order in which they were attempted and sharply discounted the utility of the second target relative to the first. In other words, the utility of hitting the first target was much larger than that of hitting the second even when hitting the second target brought much higher monetary reward. This phenomenon is called temporal discounting in economics [30], [31] but to explain our results we need to postulate large discounts over durations measured in milliseconds, a very surprising outcome. Further research is needed to determine whether the failures in time allocation we observed can be treated as a very rapid form of temporal discounting.

There are several previous studies demonstrating that human subjects can adjust the duration of *single* movements so as to nearly maximize expected gain [3]–[5]. In contrast, we find that subjects do not allocate time between *two* movements so as to maximize expected gain. A parsimonious explanation is that the motor system optimizes each component of a series of movements in isolation but not the entire series considered as a whole. Such a limitation on the complexity of movement planning is analogous to limits on reasoning and judgment associated with bounded rationality [28], [29] but in the planning of sequences of movements.

## Materials and Methods

### Apparatus

A touch monitor (Elo IntelliTouch 17 in. LCD monitor) was mounted on a Structural Framing System (McMaster Carr Inc.). The monitor was tilted to be horizontal. A double-square framing system was selected to minimize the vibration of the setup caused by the speeded pointing movement to the monitor. The experiment was run using the Psychophysics Toolbox [38], [39] on a Pentium 4 Dell Optiplex GX280. At the beginning of every experimental session, subjects completed a calibration procedure on touch location. The experimental room was dimly lit.

### Stimuli

#### Targets

The stimulus in every trial comprised a starting position (coded red) and two circular targets (coded blue, green) carrying monetary reward (Figure 1). Each target was a double-circled configuration, and we refer to the circles as the inner and outer rings. The red and blue targets were always in the same locations on the touch screen.

In the main part of the experiment (Session B below), the subject could only earn money by touching within the inner ring of a target. However, we wanted to make sure that subjects did not simply ignore one or the other target on a trial and move to touch only the other. We did so as follows. The outer ring of the target had a radius (23.5 mm) four times bigger than that of the inner ring (5.88 mm). Touches within the outer ring did not earn monetary reward but counted as an *attempt* to touch the target. As described below, we required that the subject *attempt* to touch both targets in the correct order within the time limit on every trial. If he did not do so on a trial, he would receive no reward. If he touched within the outer rings of both targets within the time limit and, in addition, touched within the inner ring of one or both targets, he received rewards as described below.

#### Events within a trial

At the beginning of each trial, the subject placed his or her right index finger on the starting position. Then the stimulus array appeared. The subject could study the stimulus array and plan his movements for as long as desired. Timing of the trial began only when the subject's index finger left the start area. The subject was required to first touch or at least attempt the blue target (referred to as target A) and then touch or attempt the green target (target B) within a fixed time period (400 ms in the main experiment).

There were two distance conditions, *equal* and *unequal*. We called the distance between the starting position and target A the “first target distance,” and the distance between target A and target B the “second target distance.” In the equal-distance trials, the first target distance and the second target distance were the same (7.5 cm). In the unequal distance trials, the first target distance remained at 7.5 cm, while the second target distance was increased to 11.2 cm.

We also manipulated the *change in movement direction* between the first and the second movement by placing target B (green) at one of eight different locations illustrated in Figure 1. Only one of the eight possible green targets was present on each trial. The eight locations were arranged such that the angle between the line joining the first and second target 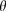 was 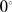, 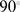 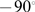, and 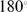 with respect to the line joining the start point and the first target. The eight targets comprised four directions and two distances, giving 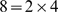 trial conditions.

#### Movement coordinate system

**Figure 9:**
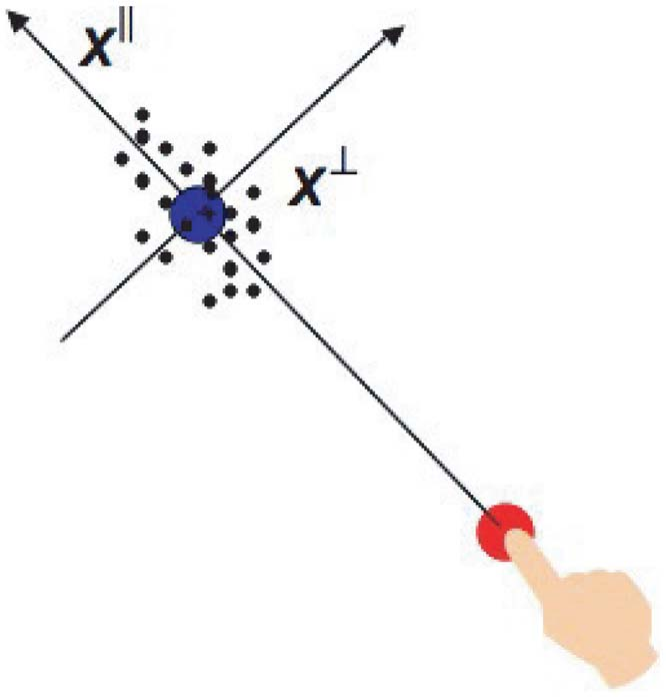
The coordinate system. We used a two-dimensional coordinate system to represent each movement. The coordinate system was embedded in the stimulus array. One axis 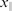 was parallel to the line connecting the start point and the end point of the movement, and the other, 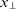 was perpendicular to the first. The origin was centered on the end point of the distribution.

For single movements, we defined a coordinate system in the plane of the touch screen whose two axes are, 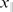 measured along the line connecting the start point of the movement and the end point and 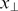 the distance perpendicular to the direction of movement (Figure 9). To allow for the possibility that accuracy changes at a different rate as a function of speed between 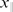 and 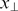, we separately modeled and estimated the standard deviation of movement end points in 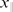, denoted as 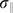 and the standard deviation of end points in 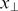, denoted as 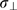, as functions of average speed.

### Procedure

#### Session A

We first trained subjects to perform the motor task. During Session A (training), the targets did not carry monetary reward. Subjects were introduced to the task in a quick warm-up session where we gave them a lenient time limit of 800 ms to complete the two movements. Each condition was run in separate blocks of trials with 10 trials per block. The order in which conditions were presented to the subjects was randomized: there were 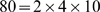 warm-up trials.

After the warm-up trials, subjects entered the formal training session where the time limit was 400 ms. They were informed of the change in time limit. The structure of formal training was the same as that for the warm-ups, except that there were 80 repetitions for each trial condition (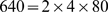 trials). During formal training, subjects started every trial by placing their index finger on the starting position and were instructed to always first touch target A followed by target B. Once the finger left the starting position, the subject had only 400 ms to attempt to touch the two targets. Subjects were instructed that, on each trial, they should (1) be sure to touch within the outer ring of each target in the specified order and (2) try to touch both targets within the inner ring.

Feedback as to which targets were touched was provided only after subjects completed both movements. Subjects received a timeout message if they failed to complete the task within 400 ms. The entire session took approximately 40 minutes to complete.

#### Session B

In this session, we assigned monetary rewards to the targets. There were two reward conditions, *equal-reward* and *unequal-reward*. In the equal-reward condition, the reward for touching the first and the second targets within the inner ring were the same (10 points). In the unequal-reward condition, the second (green) target was worth 50 points while the first target remained at 10 points. Subjects accumulated winnings over trials. The subject could receive a reward for either or both targets touched within the inner ring. However, we emphasize that, if subjects failed to touch within the outer rings of the two targets in the specified order, they received no reward for the trial. If they exceeded the time limit on a trial, they received no reward. Subjects knew that every 1000 points was worth $1 paid at the end of the experiment.

The procedure for Session B was the same as the second part of Session A (training), except that there were a total of 16 conditions (2 reward conditions x 8 trial conditions) run in separate blocks of trials. Each condition had 50 repetitions for a total of 800 trials. The entire session took approximately 80 minutes to complete.

### Subjects and Instructions

Five subjects, unaware of the purpose of the experiment, participated. Among them, three were male and two were female. All subjects were right handed and all had normal or corrected-to-normal vision. The study was approved by the Institutional Review Board at New York University. All subjects gave written informed consent prior to the experiment.

### A Model of Optimal Sequential Movement Planning

In this sequential task, the fixed time constraint induced a tradeoff of time between the two movements: the more time spent on attempting one target, the less time was available to attempt the other. Our model considers the consequences of trading off movement time between the targets on the accuracy profile of the movements. We derive the optimal tradeoff that maximizes expected gain. We emphasize that timing of each trial began only when the subject initiated movement. The subject saw the configuration on each task and had as much time as he wished to plan his movements before starting to move. Hence we need not consider a possible tradeoff between time to plan and time to move in the model.

We start by treating movements in the sequential task as a series of single and independent movements. In our data analysis, we tested whether this *independence assumption* is sensible given subjects' actual performance (see *Results* *: Movement independence*). For single movements, we used a coordinate system in the plane of the touch screen whose two axes are 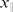 measured along the line connecting the start point of the movement and the end point and 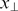 the distance perpendicular to the direction of movement as shown in Figure 8. We assume that movement endpoints 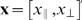
are distributed as a bivariate Gaussian random variable with mean 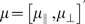 and covariance matrix
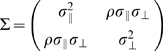
(1)where 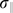 denotes the standard deviation of errors parallel to the movement direction and 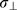 denotes the standard deviation of errors orthogonal to the movement direction. The probability distribution of movement endpoints with mean 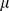 and covariance matrix 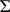 is
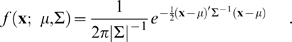
(2)


For a circular target 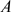 with center at 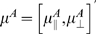 and radius *r*, the probability that the endpoints are within 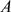 given aim point 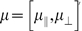 and covariance matrix 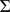 can be computed [40] as
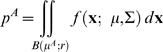
(3)where 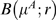 denotes the region of integration, a circular region of radius 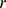 centered on 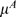 and 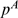 is the probability of hitting target A. We wish to emphasize that our model is readily modified to allow for off-center aiming. In the case of off-center aiming, 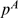 is computed by Eq. (3) by substituting 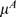 with subjects' actual mean end points.

Let *G^A^* be the gain for touching target A and 0 otherwise. Then the expected gain for the movement to target *A* is 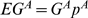. The choice of aim point 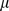 that maximizes probability and expected gain is 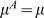, when the aim point of the movement is the center of the circular region.

The covariance matrix 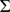 also affects 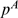 and, due to speed-accuracy tradeoff (SAT) of motor response [21], 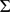 varies as a function of movement speed. We will write it and its components as functions of 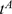, the planned movement time to target A: 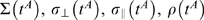. Movement time (*MT*) is a random variable and here we modeled it as 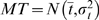 where 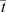 is the subject's planned movement time. The realization of a planned movement time is what we referred to as the actual movement time. In a previous study [5], we found that mean movement time was a better predictor than actual movement time. In the experiment, we estimated planned movement time, separately for each movement, of a condition by computing the mean of actual movement time across trials in that condition.

If 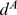 is the distance (within the touch screen) traveled during the movement, then the average speed of the movement is 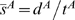. We developed a model of speed-accuracy tradeoff based on previous work. There is a large literature concerning speed-accuracy tradeoff related to Fitt's Law [22] and the linear version of it advanced by Schmidt and colleagues [24], [25]. See [23], [25], [26] for reviews. Wright and Meyer [27] investigated conditions under which each model is appropriate. In brief, the linear law describes performance at short time intervals, under conditions where visual feedback is not available and conditions when aimed movements must have precisely specified durations. Meyer et al. [23] proposed that the linear law is fundamental and developed a model of SAT in which Fitt's Law arises as a consequence of successive corrections based on visual feedback. See Meyer et al. [23] and Plamondon and Alimi [26] for reviews.

Given the short time duration of movements in our study (see Figure 7AB), the linear law based on Schmidt's work reproduced by Wright and Meyer [27] and Meyer et al. [23] is appropriate. Accordingly, we modeled the SAT based on Schmidt et al. [24], [25] as
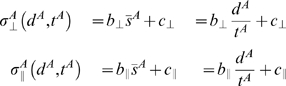
(4)where the constants 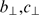, etc. characterized the SAT. We allowed for the possibility that the relation between time 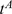 and 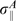 and between 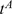 and 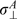 are different. We estimated the SAT separately for 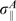 and 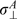 and tested them for equality. To our knowledge, we are the first to model the relation between spatial error and speed separately for the direction parallel to the movements and for the direction orthogonal to the movements. We could also allow for the possibility that 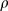 (Eq. 1, the correlation between movement errors parallel to and perpendicular to the direction of movement) changes with the duration of the movement and add a third equation to Eq. (4). However, we discovered that 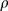 was close to 0 for all durations and conditions and can be neglected. See *Results* *: Estimating speed-accuracy tradeoff* for details.

Combining Equations (3) and (4), we can compute 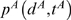 and 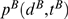 where 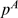 is as above and 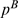 is the probability of hitting target B with the second movement. As a consequence of the independence assumption, we assume that the first movement and the second share the same mapping of time and distance to probability of touching the target (the second movement is not affected by the first other than through a tradeoff of time as detailed next). Hence we can write the awkward expressions 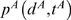 and 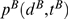 as 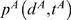 and 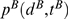 respectively. The function 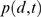 is a decreasing function of its first argument and an increasing function of its second.

In the sequential task, the fixed time limit introduced a constraint on the total movement time 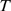, which in turn introduced a tradeoff of time between the movements. If the first movement has duration 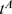 then the second movement has duration 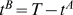. The subject has no control over the locations of targets or the distances between successive targets but he could choose the tradeoff between 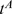 and 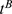. Consequently the subject's overall expected gain is
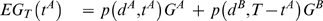
(5)


The ideal mover that maximizes expected gain would choose 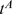 to maximize Eq. (5).

### Data Analysis

#### Movement independence

In our model, we assumed that the two, successive movements were statistically independent of one another. To examine the independence assumption, we looked at the correlation between the movement endpoints of the first and the second movements, and also looked at the conditional probabilities of hitting the second target when the first target was hit and when the first target was missed.

#### Aim-point assumption

We initially assumed that subjects aimed at the center of targets. To test the aim-point assumption, we examined, for each subject, the endpoint distribution at each target location and tested it against the hypothesis that the mean end points fell on the center of the target (Hotelling's 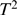 test). With sufficiently large amounts of data, it is very likely we will reject this assumption. However, if the actual deviation of subjects aim point from the center of targets is small in magnitude then the effect on the predictions of the model and its fit to the data will be correspondingly slight. Accordingly, we also compare actual probability of hitting targets to the predictions of the model with the aim-point assumption. We consider this point where we report results of tests of the assumption.

#### Estimating speed-accuracy tradeoff (SAT)

We treated movements in our sequential pointing task as a series of independent movements and assumed a linear relation between the speed and the accuracy of single movements as expressed in Eq. (4) where 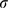 denotes spatial variability and 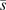 as the average speed. We assumed that the endpoint distribution is a bivariate Gaussian with a covariance matrix 
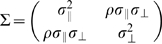
 where, as above, 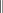 denotes ‘parallel to the movement direction’ and 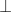 as ‘orthogonal to the movement direction’. We estimated 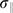 and 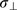 as follows. Based on the geometric relation between the first target and the second target of each direction condition (see Figure 1), we estimated 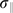 and 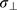 based on the endpoint variability in the parallel and perpendicular directions to the direction of movement.

Before we estimated the SAT, we needed to examine the covariance 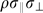 and its relation to speed. For each condition separately, we looked at the correlation 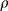 and examined whether it was significantly different from 0. If 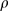 was not significantly different from 0 across different conditions, we treated the covariance as 0.

Then, to estimate SAT, we computed 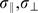 and average movement speed 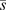 for each movement in each condition. We then regressed 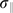 and 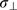 separately by 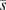 to obtain the estimated SAT. We emphasize that we estimated SAT for each direction condition separately. Hence, each direction would have its own estimated SAT profile.

The estimated SAT allowed us to compute, for a given speed of a movement, estimates of spatial variability (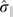 and 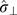) corresponding to that speed and hence the probability of hitting the first target 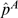 and the probability of hitting the second target 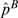, which were later used to compute the optimal solution for the tradeoff of time. After testing and failing to reject movement independence (see *Results* *: Independence assumption* below), we could combine data for both movements in estimating 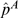 and 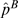. Hence, the estimated SAT reflected the relation between the speed and accuracy of a single movement independent of which movement it is.

#### Model comparison

Once we have evaluated the key assumptions in the model and obtained the direction-sensitive SAT functions for each subject, we were ready to compute the optimal solution. The optimal solution is the tradeoff of time between the two targets that maximizes Eq. (5). For each condition, we used the mean total movement time (first movement time+second movement time) as total time 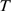 and computed the optimal tradeoff of time between the two targets and compared it with actual performance. We emphasize that dwell time was excluded when counting the first movement time and the second movement time.

#### Movement times and dwell time

For each subject, we analyzed movement times and dwell time separately for each combination of direction, distance, and reward conditions. We referred the time the finger stays on the first target before it starts moving toward the second target as *dwell time*. We reasoned that under such tight time limit (400 ms), dwell time would have little impact on how subjects prepare for the second movement. If this conjecture were true, we would expect that dwell time be constant across all conditions.
